# Would Screening for Lung Cancer Benefit 75- to 84-Year-Old Residents of the United States?

**DOI:** 10.3389/fonc.2014.00037

**Published:** 2014-03-07

**Authors:** John M. Varlotto, Malcolm M. DeCamp, John C. Flickinger, Jessica Lake, Abram Recht, Chandra P. Belani, Michael F. Reed, Jennifer W. Toth, Heath B. Mackley, Christopher N. Sciamanna, Alan Lipton, Suhail M. Ali, Richkesvar P. M. Mahraj, Christopher R. Gilbert, Nengliang Yao

**Affiliations:** ^1^Department of Radiation Oncology, University of Massachusetts Medical Center, Worcester, MA, USA; ^2^Division of Thoracic Surgery, Department of Surgery, Northwestern Memorial Hospital, Chicago, IL, USA; ^3^Department of Radiation Oncology, Pittsburgh Cancer Institute, Pittsburgh, PA, USA; ^4^Pennsylvania State University College of Medicine, Hershey, PA, USA; ^5^Department of Radiation Oncology, Beth Israel Deaconess Medical Center, Boston, MA, USA; ^6^Penn State Hershey Cancer Institute, Hershey, PA, USA; ^7^Heart and Vascular Institute, Penn State Hershey Medical Center, Hershey, PA, USA; ^8^Division of Pulmonary, Allergy, and Critical Care Medicine, Department of Medicine, Penn State Hershey Medical Center, Hershey, PA, USA; ^9^Department of Medicine, Penn State College of Medicine, Hershey, PA, USA; ^10^Department of Radiology, Penn State College of Medicine, Hershey, PA, USA; ^11^Department of Healthcare Policy and Research, Virginia Commonwealth University College of Medicine, Richmond, VA, USA

**Keywords:** lung cancer, elderly, screening, radiotherapy, thoracic surgery

## Abstract

**Background:** The National Lung Screening Trial demonstrated that screening for lung cancer improved overall survival (OS) and reduced lung cancer mortality in the 55- to 74-year-old age group by increasing the proportion of cancers detected at an early stage. Because of the increasing life expectancy of the American population, we investigated whether screening for lung cancer might benefit men and women aged 75–84 years.

**Materials/Methods:** Rates of non-small cell lung cancer (NSCLC) from 2000 to 2009 were calculated in both younger and older age groups using the surveillance epidemiology and end reporting database. OS and lung cancer-specific survival (LCSS) in patients with Stage I NSCLC diagnosed from 2004 to 2009 were analyzed to determine the effects of age and treatment.

**Results:** The per capita incidence of NSCLC decreased in the 55–74 cohort, but increased in the 75–84 cohort over the study period. Crude lung cancer death rates in the two age groups who had no specific treatment were 39.5 and 44.9%, respectively. These rates fell in both age groups when increasingly aggressive treatment was used. Rates of OS and LCSS improved significantly with increasingly aggressive treatment in the 75–84 age group. The survival benefits of increasingly aggressive treatment in 75- to 84-year-old females did not differ from their counterparts in the younger cohort.

**Conclusion**: Screening for lung cancer might be of benefit to individuals at increased risk of lung cancer in the 75–84 age group. The survival benefits of aggressive therapy are similar in females between 55–74 and 75–84 years old.

## Introduction

The results of the National Lung Screening Trial (NLST) were reported in 2011 (1). This study randomized 53,454 patients who had at least a 30-pack-year history of smoking, did not have a previous history of lung cancer, and were between ages 55 and 74 years old to receive three annual low-dose computerized tomograms (CT) or a single posteroanterior chest X-ray. Patients in the CT arm had a 20% relative reduction in lung cancer-specific mortality and a 6.7% reduction in the risk of death from any cause. These reductions appear due to finding cancers at a much earlier, more curable stage than otherwise expected (1, 2). However, this trial did not include individuals aged 75 years or older (defined as “elderly”), yet more than half of all lung cancers in North Americans occur in patients aged over 70 years (3, 4). The elderly population in the United States is increasing rapidly. Life expectancy has increased over time in all races, and the burden of lung cancer remains substantial in the elderly (5). Women aged 75 years have an average life expectancy of 12.9 years, and men have an average of 11.0 years (6).

We therefore chose to investigate whether screening might be beneficial in the elderly population (75–84 years old) by determining the outcome for patients with Stage I non-small cell lung cancer (NSCLC) in this age cohort and comparing it to that of patients 55–74 years old. Our findings suggest that individuals in both age cohorts have similar outcomes when treated in the same fashion, and therefore screening may be of benefit to elderly individuals at increased risk of lung cancer who are fit enough to undergo treatment.

## Data and Methods

### Data source

Data for this study were taken from the surveillance epidemiology and end results (SEER) program of the National Cancer Institute (NCI), which started to collect and publish cancer incidence and survival data from population-based cancer registries in 1973. The “SEER-9” registries are Atlanta, Connecticut, Detroit, Hawaii, Iowa, New Mexico, San Francisco-Oakland, Seattle-Puget Sound, and Utah. Data are available for cases diagnosed from 1973 and later for most of these registries. The “SEER-18” data-base used in this study includes the above registries and those in Los Angeles, San Jose-Monterey, Rural Georgia, Greater California, Kentucky, Louisiana, New Jersey, Greater Georgia, and the Alaska Native Tumor Registry (7). Data are available from all cases diagnosed from 2000 and later for these registries. The SEER-18 sites cover approximately 28% of the American population (8).

### Cohort selection

Since small cell lung cancer rarely presents at an early stage even when screening is employed (1–2.2%) (9), we excluded patients with this histology from our study. We included adults aged 55–84 years who were diagnosed with NSCLC in the SEER-18 data-base during 2004–2009. A total of 191,868 patients aged 55–74 years and 94,828 patients aged 75–84 years met the eligibility criteria. Since the data from the SEER registry are de-identified, no IRB approval was requested.

Outcome was examined for the 14,007 patients with NSCLC diagnosed during the years 2004–2009 for whom sufficient information was collected to assess the outcome of treatment in relation to patient and histopathologic variables. Patients included in this investigation had NSCLC as their first primary cancer, tumor size 4 cm or smaller, clinical T1-2N0 disease, extension codes 100, 110, or 300, and only one type of local treatment (e.g., patients receiving both radiation and surgery were excluded).

### Outcome variables and other covariates

The outcome variables were overall survival (OS) and lung cancer-specific survival (LCSS). Deaths from other causes were treated as censoring events. The exploratory variable of main interest was the type of treatment that patients received. Treatments were categorized as: observation only; radiation only; subtotal resection (sub-lobar resection; segmental resection, including lingulectomy; or wedge resection); and lobectomy or greater (lobectomy or bi-lobectomy, with or without extension to include the chest wall; lobectomy with mediastinal node dissection; extended lobectomy or bi-lobectomy, not otherwise specified; pneumonectomy with mediastinal node dissection; or pneumonectomy, not otherwise specified).

Other variables (in addition to age cohort) examined for their potential effect on outcome were: gender; year of diagnosis; marital status; race; Hispanic origin; tumor size; histology; grade; location; and extension. Median follow-up time was 26 and 21 months in the 55- to 74- and 75- to 84-year-old age groups, respectively.

### Statistical analysis

The incidence rates of NSCLC per 100,000 individuals in the SEER-18 population were calculated via SEERSTAT. *T*-tests were performed to analyze if there was significant difference in incidence rates by age group. Trend analyses were used to determine if incidence rates exhibit an increasing or decreasing trend over time. Chow tests were used to determine whether the slopes in two linear trend lines of incidence rates were equal by age group (10).

Chi-square and *t*-test were used to compare difference between the two age cohorts with respect to treatment, patient characteristics, and tumor characteristics. OS and LCSS were calculated using Kaplan–Meier estimation (11). The statistical significance of differences between these rates was calculated using the log-rank test. Cox proportional hazards model estimates (12) were used to show how treatment and other covariates were related to outcome. The older cohort was divided into two age groups in the multivariate analyses (aged 75–79 and 80–84 years). The hazards ratio (HR) for treatments and their corresponding *p*-values were estimated from the regression coefficient, and the standard error from the proportional hazards models.

To better understand the relationship of treatment and survival between the age cohorts, we included an interaction effect between treatment and age group in proportional hazards models. All multivariate analyses were conducted using SAS software version 9.2, and all statistical tests assumed a two-tailed α = 0.05.

## Results

**Figure 1:**
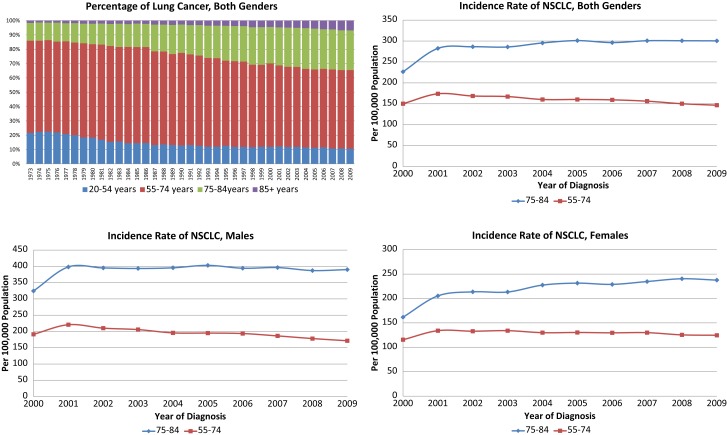
**Incidence and proportions of non-small cell lung cancer, 2000–2009 in both genders, females and males**.

The annual incidence rates per 100,000 persons for NSCLC were significantly higher in the 75–84-year-old age group than in the younger age group (Figure 1). Of note, the annual incidence rates increased over time for the older female age cohort (*p* = 0.0017) while staying stable for older males and younger females and decreasing for younger males (*p* = 0.0065). The Chow tests revealed significant difference in the slopes of trend lines (*p* = 0.0017), especially for women (*p* = 0.0011). The proportion of NSCLC cases fell in the 55–74 group and increased in the 75–84 group during the study period for all stages as well as Stage I tumors ≤4 cm (data not shown).

Characteristics of the 14,007 patients who met our study’s eligibility criteria for outcome analysis (9,588 in the younger and 4,419 in the older cohorts) are listed in Table 1. The study cohort was evenly distributed during 2004–2009, and the yearly distributions were not significantly different in the two age groups. The proportion of widowed patients in the younger group was substantially lower than in the older group (13.7 vs. 33.5%; *p* < 0.0001); 54.1% of patients in the younger group were female, which was lower than the older group (56.7%, *p* = 0.0049); and 85.7% of patients in the younger group were white, lower than in the older group (89.4%, *p* < 0.0001). Approximately 96% of patients were non-Hispanic, and the distributions of Hispanic ethnicity were not significantly different in the two age groups. There were fewer squamous cell carcinoma patients in the younger group than in the older group (28.8 vs. 33.1%, *p* < 0.0001); and 54.1% of the tumors in the younger group were well-differentiated or moderately differentiated, higher than among patients in the older group (50.7%, *p* < 0.0001). Approximately 28% of patients had cancer diagnosed in the left upper lobe, and the distributions of location were not significantly different in the two age groups. The average tumor size was 1.4 mm smaller in the younger group than the older group (19.1 vs. 20.5 mm, *p* < 0.0001). As expected, younger patients were more likely to be treated with lobectomy or pneumonectomy (67.3 vs. 50.5%, *p* < 0.0001).

**Table 1:** **Patient characteristics by age groups (*N* = 14,007)**.

	55–74	75–84	*p*-Value
**TREATMENT**			
Observation	612 (6.4)	541 (12.2)	<0.0001
Radiation	903 (9.4)	829 (18.8)	
Subtotal resection	1,621 (16.9)	818 (18.5)	
Lobectomy	6,452 (67.3)	2,231 (50.5)	
**YEAR AT DIAGNOSIS**			
2004	1,476 (15.4)	659 (14.9)	0.6155
2005	1,468 (15.3)	689 (15.6)	
2006	1,620 (16.9)	726 (16.4)	
2007	1,661 (17.3)	735 (16.6)	
2008	1,664 (17.4)	788 (17.8)	
2009	1,699 (17.7)	822 (18.6)	
**MARITAL STATUS**			
Married	5,618 (58.6)	2,203 (49.9)	<0.0001
Separated	91 (1.0)	16 (0.4)	
Single (never married)	989 (10.3)	245 (5.5)	
Widowed	1,315 (13.7)	1,482 (33.5)	
Unknown	281 (2.9)	135 (3.1)	
**GENDER**			
Female	5,189 (54.1)	2,504 (56.7)	0.0049
Male	4,399 (45.9)	1,915 (43.3)	
**RACE**			
White	8,215 (85.7)	3,951 (89.4)	<0.0001
American Indian/Alaska native	34 (0.4)	10 (0.2)	
Asian or Pacific Islander	459 (4.8)	221 (5.0)	
Black	848 (8.8)	227 (5.1)	
Other unspecified (1991+)	7 (0.1)	4 (0.1)	
Unknown	25 (0.3)	6 (0.1)	
**HISPANIC ORIGIN**			
Non-Spanish–Hispanic–Latino	9,209 (96.1)	4,241 (96.0)	0.8324
Spanish–Hispanic–Latino	379 (4.0)	178 (4.0)	
**HISTOLOGY**			
Squamous	2,411 (28.8)	1,295 (33.1)	<0.0001
Adenocarcinoma-BAC	780 (9.3)	282 (7.2)	
Large cell	310 (3.7)	139 (3.6)	
Adenocarcinoma	3,956 (47.3)	1,651 (42.1)	
Other NSCLC	177 (2.1)	78 (2.0)	
NSCLC NOS	727 (8.7)	473 (12.1)	
**GRADE**			
Well-differentiated	1,537 (16.0)	677 (15.3)	<0.0001
Moderately differentiated	3,648 (38.1)	1,563 (35.4)	
Poorly differentiated	2,705 (28.2)	1,148 (26.0)	
Undifferentiated; anaplastic	165 (1.7)	76 (1.7)	
Unknown	1,532 (16.0)	955 (21.6)	
**LOCATION (%)**			
Left lower lobe	1,214 (12.7)	618 (14.0)	0.0545
Right lower lobe	1,565 (16.3)	743 (16.8)	
Main bronchus	15 (0.2)	10 (0.2)	
Left upper lobe	2,637 (27.5)	1,241 (28.1)	
Middle Lobe	496 (5.2)	227 (5.1)	
Overlapping lesions	41 (0.5)	21 (0.5)	
Right upper lobe	3,434 (35.8)	1,460 (33.0)	
Left, NOS	64 (0.7)	42 (1.0)	
Right, NOS	71 (0.7)	40 (0.9)	
NOS	51 (0.5)	17 (0.4)	
Median tumor size, mm	19.1 (6.5)	20.5 (6.3)	<0.0001

*Values are *N* (%) or median (standard error)*.

*Chi-square tests and *t*-tests*.

*Some values missing*.

**Figure 2:**
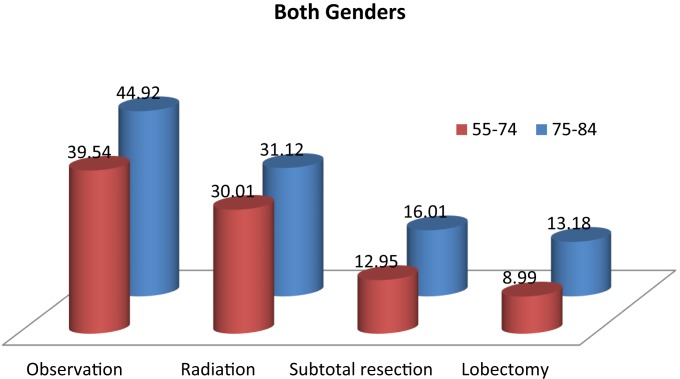
**Cause of death from lung cancer (crude) by treatment and age group (%): 2004–2009**.

Table 2 and Figure 2 show the proportion of NSCLC patients who died (crude death rates) from lung cancer by treatment and age group during 2004–2009. Lung cancer was the most common cause of death in all treatment groups in the younger age cohort. Lung cancer was also the most common cause of death in all treatment groups in the older cohort. Crude death rates from lung cancer decreased in both age cohorts as the aggressiveness of treatment increased.

**Table 2:** **Top three causes of death and 5-year overall survival rates in patients with stage I non-small cell lung cancer, 2004–2009**.

	55–74 Age group				75–84 Age group			
	Observation	Radiation	Subtotal resection	Lobectomy	Observation	Radiation	Subtotal resection	Lobectomy
Sample *N*	612	903	1,621	6,452	541	829	818	2,231
Alive %	40.0	54.3	77.9	84.1	33.8	53.4	68.3	74.7
Death from lung cancer %	39.5	30.0	13.0	9.0	44.9	31.1	16.0	13.2
Diseases of heart %	4.7	2.8	1.4	1.7	5.2	4.3	5.1	2.8
Chronic obstructive pulmonary disease and allied cond %	5.1	5.2	2.2	1.1	3.7	4.0	3.2	1.8
**SAMPLE** ***N***								
Male	299	421	748	2,931	234	342	359	980
Female	313	482	873	3,521	307	487	459	1,251
**DIED OF LUNG CANCER**								
Male %	43.5	32.8	13.8	10.5	43.2	30.7	19.5	16.0
Female %	35.8	27.6	12.3	7.7	46.3	31.4	13.3	11.0
**5-YEAR OVERALL SURVIVAL**								
Male %	10.5	22.4	59.2	69.6	8.8	13.0	34.2	50.8
Female %	25.0	28.7	61.7	75.7	10.9	19.8	57.9	64.2

**Figure 3:**
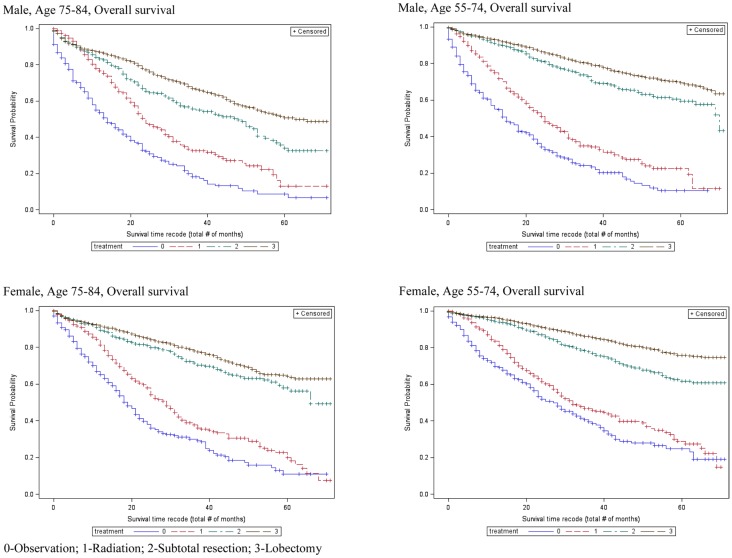
**Overall survival curves for patients by age groups**.

Table 2 also shows that the 5-year OS rates improved significantly with increasingly aggressive treatment in both the 55–75 and 75–84-year age groups. The survival curves in Figure 3 again revel that OS improved significantly with increasingly aggressive treatment in the 75–84 group among both genders. The survival curves in the older group for each treatment appear to be similar to those for the younger group.

Adjusted risks of death were determined using standard multivariate Cox proportional hazards models, including year of diagnosis, marital status, race, Hispanic ethnicity, tumor size, tumor grade, tumor location, histology, tumor extension, and treatment covariates. Table 3 displays the predictors of OS and LCSS from the hazards models in males and females in three age groups (the elderly group was split into 75- to 79- and 80- to 84-year age groups). Similar to the younger cohort, the risk of death due to any cause in the 75–79 and 80–84-year age group was significantly higher in patients treated with subtotal resection, radiation, or observation than for patients treated with lobectomy or greater.

**Table 3:** **Adjusted hazards ratios for survival among the elderly age group: comparison between treatments**.

	Overall survival	Lung cancer-specific survival
**55–74-YEAR AGE GROUPS**		
Male	*N* = 3,951	*N* = 3,951
Observation	6.090 (<0.0001)	7.285 (<0.0001)
Radiation	3.781 (<0.0001)	4.652 (<0.0001)
Subtotal resection	1.387 (0.0003)	1.401 (0.0055)
Lobectomy (reference)	1.000	1.000
Female	*N* = 4,301	*N* = 4,301
Observation	5.497 (<0.0001)	6.170 (<0.0001)
Radiation	3.487 (<0.0001)	3.838 (<0.0001)
Subtotal resection	1.697 (0.0271)	1.789 (<0.0001)
Lobectomy (reference)	1.000	1.000
**75–79-YEAR AGE GROUPS**		
Male	*N* = 1,087	*N* = 1,087
Observation	3.940 (<0.0001)	5.302 (<0.0001)
Radiation	2.008 (<0.0001)	2.665 (<0.0001)
Subtotal resection	1.325 (0.0451)	1.340 (0.1405)
Lobectomy (reference)	1.000	1.000
Female	*N* = 1,324	*N* = 1,324
Observation	6.268 (<0.0001)	10.283 (<0.0001)
Radiation	2.862 (<0.0001)	3.962 (<0.0001)
Subtotal resection	1.420 (0.0221)	1.533 (0.0389)
Lobectomy (reference)	1.000	1.000
**80–84-YEAR AGE GROUPS**		
Male	*N* = 631	*N* = 631
Observation	3.862 (<0.0001)	3.955 (<0.0001)
Radiation	1.999 (0.0003)	2.261 (0.0011)
Subtotal resection	1.878 (0.0005)	1.672 (0.0424)
Lobectomy (reference)	1.000	1.000
Female	*N* = 827	*N* = 827
Observation	4.459 (<0.0001)	5.874 (<0.0001)
Radiation	3.276 (<0.0001)	4.653 (<0.0001)
Subtotal resection	1.137 (0.5387)	1.090 (0.7816)
Lobectomy (reference)	1.000	1.000

*Adjusted for year of diagnosis, marital status, race, Hispanic ethnicity, tumor size, tumor grade, tumor location, histology, and tumor extension*.

Multivariate analysis included an interaction effect between treatment and age group showed that there are gender differences in how aggressive treatment affects outcome for patients in different age cohorts (Table 4). For female patients, the survival benefits of aggressive therapy are similar between 55–74 and 75–84 year-old age groups. In contrast, the survival benefits of aggressive therapy are different between the 55–74 group and the 75–84 group in male patients.

**Table 4:** **Wald tests of the interaction effect between age group and treatment in Cox proportional hazards model**.

	*N*	Wald chi-square	Pr > Chi Sq
Overall survival, male, 55–74 vs. 75–79	5,038	17.3800	<0.0001
Lung cancer-specific survival, male, 55–74 vs. 75–79	5,038	6.6519	0.0839
Overall survival, male, 55–74 vs. 80–84	4,582	29.1234	<0.0001
Lung cancer-specific survival, male, 55–74 vs. 80–84	4,582	14.5713	0.0022
Overall survival, female, 55–74 vs. 75–79	5,625	6.4545	0.0915
Lung cancer-specific survival, female, 55–74 vs. 75–79	5,625	8.1917	0.0422
Overall survival, female, 55–74 vs. 80–84	5,128	7.6409	0.0540
Lung cancer-specific survival, female, 55–74 vs. 80–84	5,128	4.8190	0.1855

*Adjusted for year of diagnosis, marital status, race, Hispanic ethnicity, tumor size, tumor grade, tumor location, histology, tumor extension, age group, and treatment*.

Causes of mortality and death rates within 90 days of treatment are listed in Table 5. The mortality rates within the observation arms exceeded those of the active treatment arms for both age group categories.

**Table 5:** **Mortality rates and causes of mortality within 90 days of treatment**.

	55–74 Group				75–84 Group			
	Observation	Radiation	Sub-lobar	Lobectomy	Observation	Radiation	Sub-lobar	Lobectomy
Initial patient #	612	903	1,621	6,452	541	829	818	2,231
% Mortality 30 days	8.5	0.8	1.3	1.0	9.6	1.7	1.8	2.1
% Mortality in 31–90 days	7.4	2.8	1.0	1.4	5.1	2.5	3.5	3.5
**CAUSES OF MORTALITY IN FIRST 90 DAYS^a^**								
Lung cancer (%)	52	84	46	53	48	64	24	50
Heart disease (%)	14		11	12	17	12	26	10
COPD and related conditions (%)	9		6		7	6	17	9
Suicide and self-inflicted injury (%)		6						
Unknown (%)	7		11	13		12	7	14
Pneumonia and influenza (%)							7	6
Other infectious diseases (%)			6					
CVA (%)							5	

*^a^ Only causes of death exceeding 5.0% were listed for each treatment and age group category*.

## Discussion

After the NLST trial report appeared, an expert panel composed of members of the National Comprehensive Cancer Network (NCCN), American College of Chest Physicians, American Society of Clinical Oncology, and American Cancer Society reviewed the literature and endorsed screening in patients aged 55- to 74-years who have a 30-pack-year history of smoking who continue to smoke or quit smoking within the past 15 years (13). However, the American Association for Thoracic Surgery recommended screening for smokers and former smokers with a 30-pack-year history of smoking and long-term lung cancer survivors aged 55–79 years (4). The NCCN has recommended screening according to risk criteria starting at the age of 50, but did not recommend an upper age limit (14). Elderly patients were not included in four prospective, randomized trials investigating the role of low-dose CT screening (15–18). Although three trials included patients older than the NLST [maximum age 75, 76, and 80 years, respectively (19–21)], all are much smaller and have not reported an effect of CT screening on LCSS and OS. This is unfortunate, since the elderly make up a rapidly increasing part of the population of the United States and other industrialized countries, and their incidence rate of lung cancer is higher than for younger age groups.

The role of aggressive treatment for lung cancer in elderly patients has been controversial. Clearly some patients who might be eligible for a screening program based on smoking history will not receive either radiation or surgery because of refusal or co-morbidities. Additionally, smoking-related co-morbidities and quality of life worsen in the elderly smoking population as compared to younger patients (22). However, in our investigation, lung cancer remains the most common cause of death for patients in this age group who develop this diagnosis, and aggressive treatment seemed to benefit those who underwent it (particularly for women). Moreover, lung cancer deaths remain the most common cause of death despite the inclusion of only Stage I tumors and without the exclusion of patients with multiple co-morbidities. Additionally, despite the broad spectrum of treating physicians in SEER, the 90-day mortality remained low (<6%) in all active treatment arms, suggesting appropriate candidate selection. Because the majority of patients receive a definitive surgical procedure in the younger and older populations (84.2 and 69.0%, respectively), we assume that like past studies (23, 24), those patients not selected for surgery most likely were medically inoperable. It should be emphasized that even in this unselected population, the majority of the elderly population with Stage I NSCLC were able to receive surgery, the standard of care, with relatively low rates of mortality (30- and 31- to 90-day mortalities were 2.1 and 3.5% in the lobectomy group and 1.8 and 3.5% in the sub-lobar resection group) during the post-operative time period.

As a society, we must be concerned with the costs of screening as well as the radiation exposure in the patients undergoing screening. Nevertheless, low-dose CT screening could also be used to detect other smoking-related ailments such as coronary artery disease, chronic pulmonary disease, and osteoporosis (25) as well as other smoking-related cancers (26). Furthermore, because radiographic signs associated with COPD (pulmonary artery enlargement and percentage of lung with a density of ≤−950 Hounsfield units) (27, 28) are associated with acute COPD and changes in FEV1, such changes could be used for evaluation and treatment.

There are many limitations to the SEER database. It does not include information concerning co-morbidities, past or present cigarette use, type of radiotherapeutic treatment [stereotactic body radiation therapy (SBRT) or conventional external beam], family history of cancer, medications, chemotherapeutic treatments, occupational exposures, symptoms of lung cancer, and recent weight loss. Additionally, our patient population is predominantly Caucasian, therefore, our results may not pertain to other racial groups. Nonetheless, we feel that the findings from our study are provocative.

The success of any screening program depends upon the ability to find early-stage disease and whether treatment of early-stage disease is beneficial. Because lung cancer survival depends greatly upon initial tumor stage (29) and only small improvements in survival have been seen in the last several decades in advanced disease (30), we feel that our study may help identify a population who were not identified in the initial screening studies and who may benefit from lung cancer screening. Although, screening may result in unnecessary treatment for breast and prostate cancers, our results show that even in Stage I tumors (4 cm or less in size) almost 40% of patients in both the younger and older groups will succumb to lung cancer if they do not receive radiation or surgery. Additionally, despite the expected increase in smoking-related co-morbidities with age (22), the majority of the elderly population received surgical treatment and had an increase in survival as the treatment became increasingly aggressive similar to the younger patient group who would be eligible for screening. Furthermore, lung cancer is the most frequent cause of cancer death in both genders (31).

It should be noted that our results demonstrate an increasing incidence of lung cancer, and beneficial effects from aggressive treatment in the 75- to 84-year-old age group, but they do not suggest a screening population *per se*. Like the NLST, we eliminated all patients with previous lung cancer and information concerning co-morbidities was not available. Additionally, co-morbidity data within large administrative databases depend upon the accuracy of coding which has been noted to be subject to much variability and underreporting in the past (32–34). However, differently than the NLST, smoking history was not known. Therefore we could not limit our analysis to patients with a 30-pack-year history of smoking. Nevertheless, because greater than 85% of lung cancers in the US are caused by cigarette smoking (35), the majority of patients in our study were most likely current or past cigarette smokers. Furthermore, even within the NLST, it appears the further refinement of eligible patients would result in a more optimal selection of candidates for screening. Sixty percent of patients at highest risk for lung cancer death in 5 years accounted for 88% of screening-prevented lung cancer deaths. These authors noted similar results when assessing the benefits of screening according to lung cancer incidence and that both the estimate of lung cancer death and incidence increased with age (36). Therefore, we feel that prospective studies are needed to assess the most beneficial populations to screen for lung cancer, but we do not feel that patients should be discriminated against screening based upon age alone.

We feel that the beneficial effects of treatment may have been underestimated in our patient population. SEER-18 represents approximately 28% of the US population regardless of physician expertise or hospital volume. Because lung cancer surgery depends greatly upon both hospital (37) and physician volume (38), the surgical outcomes may not be optimized. Additionally, SBRT has higher control rates than conventional radiotherapy and may offer an improvement in survival and control rates similar to surgical resection (24, 39). However, as of 2007, only 1.1% of the patients with Stage I NSCLC in the Medicare-SEER population received SBRT as compared to 14.8% who received conventional radiotherapy (40). Therefore, the full beneficial effects of radiotherapy are probably under appreciated in our investigation. Moreover, SBRT can be easily administered to patients with multiple co-morbidities and may result in fewer patients being observed (39).

## Conclusion

Because the rates of lung cancer are rising in the elderly and because increasingly aggressive treatment is beneficial in these patients, screening the 75- to 84-year-old age groups may be beneficial. Furthermore, it should be noted that most of this unselected, elderly population was able to undergo a definitive surgical resection. As recently shown in patients who were eligible for the NLST, even in the 55- to 74-year-old age group, further refinement of the at-risk patient populations is needed to find who would benefit most from screening (33). We feel that patients 75 and older should not be discriminated against lung cancer screening based upon age alone.

## Author Contributions

Data acquisition: Nengliang Yao, John M. Varlotto; Data analysis: John M. Varlotto, Jessica Lake, John C. Flickinger, Nengliang Yao; Manuscript writing: Abram Recht, John M. Varlotto, Nengliang Yao; Final approval: Abram Recht, John M. Varlotto, Nengliang Yao, Malcolm M. DeCamp, John C. Flickinger, Jessica Lake, Chandra P. Belani, Michael F. Reed, Jennifer W. Toth, Heath B. Mackley, Christopher N. Sciamanna, Alan Lipton, Suhail M. Ali, Christopher R. Gilbert, and Richkesvar P. M. Mahraj. Guarantor of the entire manuscript: John M. Varlotto.

## Conflict of Interest Statement

The authors declare that the research was conducted in the absence of any commercial or financial relationships that could be construed as a potential conflict of interest.
